# Neuropathic phenotypes and natural history of FAP

**DOI:** 10.1186/1750-1172-10-S1-I2

**Published:** 2015-11-02

**Authors:** David Adams

**Affiliations:** 1 Centre Paris-Sud, APHP, Hopital de Bicetre and Centre de Reference National des Neuropathies Amyloides Familiales, 94275 Le Kremlin-Bicetre, France

## 

TTR-FAP have been described more than 60 years ago by Corino Andrade in Porto (Brain, 1952). This peculiar disease affected many families with an autosomal dominant transmission, in the third decade of life, characterized by a progressive peripheral neuropathy starting in the lower extremities with initial impairment of thermal and pain sensibilities associated with gastrointestinal disorders and loss of weight. Natural history has been reported by Paula Coutinho (1980) with progression in 3 stages of the disease : stage 1, a disease limited to the lower limbs and a patient is still walking without any help (5.6 years), stage II progressive motor loss in the lower limbs with steppage needing help for walking (4.8 years), and a stage III in which the patient is bedridden or confined to a wheelchair (2.3 years). Death comes within an average time of 10.8 years. Late onset (LO>50 years) cases of V30M were reported in the 2000’s, with a different phenotype marked by all sensory modalities involvement, relatively mild autonomic symptoms, predominantly in male patients and sporadic cases (50%). Three new phenotypes have been described in late onset cases more recently: upper limb onset neuropathy, ataxic neuropathy and motor neuropathy. In LO V30M, there is an earliest and rapid impact on locomotion, need for aid for walking within 3 years from onset, and a shorter survival 7.3 years (Koike et al, JNNP 2012). There is genotype impact on severity and progression of the neuropathy and a correlation of Neuropathy Impairment Scores (NIS) and locomotion score PND (Adams et al, Neurology 2015 ; Mariani et al, in press). The phenotype variety, frequent absence of family history and autonomic dysfunction in late onset cases contribute to delay diagnosis. The early and increase uptake of TTR gene testing should allow to increase the identification of TTR-FAP.

